# May Argyrophilic Nucleolar Organizer Regions Be Used as a Biomarker for the Detection of the Degree of Ischemic Damage Instead of Tunel in Testicular Torsion?

**DOI:** 10.3390/medicina57111177

**Published:** 2021-10-29

**Authors:** Murat Kabaklıoğlu, Recep Eroz, Murat Kaya

**Affiliations:** 1 Department of Pediatric Surgery, Medical Faculty, Duzce University, Duzce 81620, Turkey; murat.kaya@duzce.edu.tr; 2 Department of Medical Genetics, Medical Faculty, Aksaray University, Aksaray 68000, Turkey; receperoz@duzce.edu.tr

**Keywords:** testis, ischemia, nucleolar organizer regions, rDNA, AgNOR

## Abstract

*Background and Objectives*: It is of great importance to obtain information about the severity of ischemic damage and duration of testicular torsion for an effective treatment strategy. Nucleolar-organizing regions (NORs) are sites of the ribosomal genes composed of ribosomal DNA and proteins. Post-silver staining NORs are termed “AgNOR”. Since AgNORs clearly reveals the self-renewal potential of cells damaged in ischemic events, we performed the current study. *Materials and Methods*: The study was carried out in four groups as control, sham, early, and late T/D. In the surgical groups, testes were corrected after a 4-h ischemia period. Testicular tissue samples were taken on the third day after detorsion in group 1, 2, 3, and on the tenth day after detorsion in group 4. TUNEL and silver stainings were applied to all samples. *Results:* The differences were significant among the groups for both mean AgNOR number and total AgNOR area/total nuclear area (TAA/TNA). Moreover, the differences between control and early torsion-detorsion (T/D), between control and late T/D, between sham and early T/D, between sham and late T/D, and between early T/D and late were statistically significant for AgNOR amount. Furthermore, statistically significant differences among the groups for an average number of apoptotic cells per tubule and the percentage of apoptotic tubule values were detected. *Discussion*: The apoptotic index gives the ratio of cells that are damaged and will die in a programmed way and cells that remain intact, rather than show the viability of the returning testicle. However, by measuring cells that regenerate with AgNOR, we can show not only those that survive but also cells that can repair themselves. *Conclusion*: AgNOR proteins are usable for the early observation of ischemic injury levels. The amount of AgNOR protein can enlighten us about the extent of testicular damage after T/D treatment. It may also help the physician in the development of effective treatment strategies for cases.

## 1. Introduction

Acute scrotum is encountered frequently by pediatric surgeons/urologists in pediatric emergency departments. Testicular torsion, which involves twisting of all testicles and testicular structures, differs from other acute scrotum causes, especially in terms of urgency in treatment. There are two types of testicular torsion diagnosed in boys at 3.8 out of 10^5^ each year: supravaginal torsion seen in the neonatal period and infancy, and intravaginal torsion in adolescence [1]. Unfortunately, the rate of orchiectomy in boys operated for testicular torsion is 42% and responsible for about 10% to 15% of emergency ailments of the scrotum in patients under 18 years of age [2].

Surgical correction in torsion treatment should be done urgently and in good time because the degree and duration of the torsion increase the complication rate and even result in atrophy. However, even if the life supports of the testis come back, damage may occur with an unknown mechanism that researchers have been trying to prevent with various agents for a long time. Since the blood flow is terminal, testicles are very sensitive to ischemic damage because the testicular arteries cannot form anastomosis and the inelastic structure of the tunica vaginalis restricts compensatory expansion. Due to the increased permeability of the blood-testicular barrier, the altered semen quality and reproductive ability may not return to normal even after the blood flow is successfully restored. So, the pathophysiology of testicular torsion-detorsion (T/D) focuses on acute ischemia-reperfusion (I-R) damage to the testicle, including the ischemic duration of the torsion and reperfusion injury of the detorsion, that may result in a possible impairment in fertility [3].

For several decades, detailed research has been conducted to find effective methods and substances used for medical treatment to reduce or even prevent testicular I-R damage. However, so far, no other strategy has been successfully applied clinically, including sub-scrotal ice application [4]. The complex pathophysiology mechanism that causes testicular I-R injury remains a mystery for human beings. However, ROS formed during I-R has been demonstrated to be a momentous actor of this process. It is certain that there is excessive toxic substance production in the microcirculation of many different tissue types due to I-R damage. Moreover, during reperfusion, vascular endothelial cell damage and microcirculation irregularity may occur, resulting in organ dysfunction [5,6]. This damage to germ cells ultimately leads to apoptosis.

One of the most important indicators of testicular damage after ischemia is the apoptotic index. The apoptotic index is determined by the ratio of the number of apoptotic cells to viable cells. For this, first of all, apoptosis must be determined by making it visible in cells. For this purpose, various morphological and biochemical methods have been developed. DNA fragmentation can be determined by histochemical (TUNEL) and biochemical (agarose gel electrophoresis) methods and ELISA. Transmission electron microscopy is a suitable method for morphological changes. Among these, TUNEL method has become the standard in the evaluation of apoptotic index [7]. However, the disadvantage of the TUNEL method is to be long, complicated, and partly subjective in the evaluation part.

Nucleolar-organizing regions (NORs) are the site of the ribosomal genes composed of ribosomal DNA (rDNA) and proteins, which are transcribed into ribosomal RNA, processed and becoming a part of mature ribosomes. Some of them are argyrophilic features. Post-silver staining NORs can be readily recognized as especially localized black spots along with the nucleolar space and are termed “AgNOR”. Various studies about the importance of the interphase AgNOR quantity in different cells were done [8,9,10,11,12,13,14,15,16,17,18,19,20]. The most reliable results may be obtained using the measurements of protein amounts by software program for cytometric purpose. Therefore, the using of a software program designed for the evaluation of the AgNOR (especially TAA/TNA ratio) can be minimized subjectively.

AgNOR detection with silver staining is an efficient, reliable, cheap, and sensitive method in the evaluation of ischemia damage due to the evaluation of a specific function in cell metabolism, the ability to evaluate a large number of samples in a short time and economically, to achieve quantitative and comparable results, and to standardize test methods. Since AgNORs are a good indicator of the proliferation index, it clearly reveals the self-renewal potential of cells damaged in ischemic events [8,9,10,11,12,13,14,15,16,17,18,19,20].

To the best of our knowledge, there is no study about the evaluation of mean AgNOR number and total AgNOR area/total nuclear area (TAA/TNA) ratio in testicular torsion/detorsion process. So, we performed the current study to detect whether AgNOR methods can be used instead of the classical TUNEL method and compare the classical TUNEL method used for this purpose with the AgNOR method.

## 2. Materials and Methods

### 2.1. Experimental Design and Animal Groups

The realization of this experimental study was possible after the approval of the local Animal Experiments Ethics Committee of Düzce University (7 April 2020). The study was performed on mature male albino Wistar rats (*n* = 20) weighing 200–300 g. Rats were housed at Düzce University Experimental Animal Application and Research Center, in plastic cages, in a temperature-controlled room (21–22 °C), alternating with natural light and darkness for 12 h each time, and animals were allowed to feed freely water and food. The rats were indiscriminately separated into four groups each including 5 rats:

Group I: The control group;

Group II: The sham group, consisting of animals that have gone through all surgical stages but whose testicles are not torsioned (orchiectomies were performed three days later);

Group III: Early T/D group: (orchiectomies were performed three days later); 

Group IV: Late T/D group: Testicular T/D surgery was performed on rats in this group such as group III but the orchiectomies were carried out 10 days later.

### 2.2. Experimental Testicular T/D Procedure

All procedures applied to animals were performed under sterile conditions in accordance with the surgical rules with xylazine (10 mg/kg i.p.) and ketamine (90 mg/kg, i.p.) anesthesia. In all torsion groups, the tunica vaginalis was opened with a midline scrotal incision, after reaching the right testicle, it was torsioned by turning it 720° clockwise and fixed to the scrotum with a 5/0 silk suture to maintain its position [21]. At the end of the 4th hour when the testis remained ischemic, detorsion was performed by rotating it counterclockwise and returned to its natural position before the surgical procedure. Using the same anesthesia, testicular tissue samples were taken on the third day after detorsion in groups 1, 2, 3, and on the tenth day after detorsion in group 4.

### 2.3. Evaluation of Germ Cell Apoptosis

**Figure 1:**
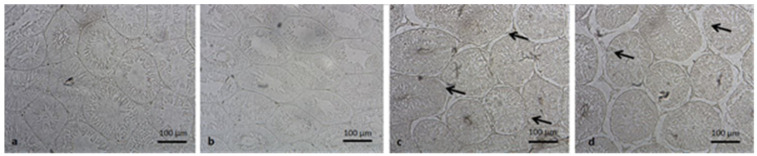
Demonstrative examples of TUNEL staining in Seminiferous tubules of each group. (**a**): control; (**b**): sham; (**c**): ET/D (early torsion/detorsion group); (**d**): LT/D (late torsion/detorsion group).

Testicular samples of each experimental group were individually fixed in 4% paraformaldehyde solution with Phosphate buffered saline solution, after enumerating without the knowledge of the investigator. After the fixation process was completed, the dehydration step of the testicles was performed with ethanol and xylene and after being blocked by embedding in paraffin, they were cut into 4–5 µm sections. In order to detect apoptosis, apoptotic nuclei in tissue sections were identified by in situ terminal deoxynucleotidyl transferase-mediated deoxyuridine triphosphate-biotin notch end labeling (TUNEL) method [22]. In situ cell death detection kit (ApopTag^®^ Plus Peroxidase In Situ Apoptosis Kit, Elabscience Houston, TX, USA) was used in accordance with the manufacturer’s instructions for use and methyl green paint was applied for the counterstaining. Under light microscopy (400× magnifications), approximately one hundred seminiferous tubule cross-sections from each specimen were appraised for the appearance of apoptotic nuclei by counting manually the positively stained nuclei. TUNEL analyses were expressed by an average number of apoptotic cells per tubule (ANPCT) and the percentage of apoptotic tubule values (PAT) (Figure 1).

### 2.4. AgNOR Staining

The samples of testicular tissue (with dimensions of about 1 × 1 × 1 cm^3^) were embedded in paraffin blocks and 4-μm sections were taken. The tissue sections of testes were deparaffinized in xylene and rehydrated in graded alcohol solutions. After re-hydration, the slides were air-dried (for 15 min) and fixed in absolute methanol for 5 min. Then all slides were silver stained with slight modification of Benn and Perle protocol [23] and Lindner [24]. For this purpose, the solution made by mixing one volume of 2% gelatin in 1% aqueous formic acid and two volumes of 50% silver nitrate was dropped on the slides and incubated at 37 °C for fifteen min in the dark. Then the slides were rinsed with bi-distilled water.

### 2.5. Image Analysis of Mean AgNOR Number and Total AgNOR Area/Total Nuclear Area

(TAA/TNA) ratio: Fifty nuclei per slide were evaluated. Silver-stained testes cells of each rat were photographed using a light microscope (Eclipse 80i; Nikon, Tokyo, Japan) via digital camera attachment (Digital Sight DS-Fi1c; Nikon, Tokyo, Japan) and evaluated using ImageJ version 1.47 t image processing software (National Institude of Mental Health, University of Wisconsin, MD, USA) [25]. The mean AgNOR number was detected by counting and the TAA/TNA ratio was detected using the “freehand selection” tool for each nucleus. The same testicular tissue samples were used for TUNEL and AgNOR staining.

In our studies, 50 cells were evaluated for each rat such as our previously published studies on the current topic. In the current study, we included 20 rats. So, a total of 1000 cells (250 cells for each group) were evaluated using the computer-assisted program Image J. ImageJ has a large and knowledgeable worldwide user community. More than 1700 users and developers subscribe to the ImageJ mailing list. The tasks may be automated and custom tools may be created using macros. More than 300 macros are available on the ImageJ Web site. Area, mean, standard deviation, min and max of selection, entire image, lengths, and angles may be measured. Real-world measurement units such as millimeters are used (https://imagej.nih.gov/ij/features.html, (accessed on: 28 September 2021)). In the current study, fifty nuclei per slide were evaluated.

### 2.6. Statistical Analysis

The data were evaluated using the Statistical Package for Social Sciences (IBM Corp.; Armonk, NY, USA) for Windows 22.0. The Shapiro–Wilk test was used to detect the distribution of data. Because the data were not normally distributed (*p* < 0.05), non-parametric tests were used for statistical analysis. In addition to descriptive statistic (number, mean, standard deviation (SD), median and range), Kruskal–Wallis test was used for the comparison of all groups and Mann–Whitney U tests were used for pairwise comparison of groups, respectively. Moreover, polynomial regression test was performed. The *p* < 0.05 was accepted as statistically significant.

## 3. Results

In addition to mean AgNOR number and mean TAA/TNA ratio of each subgroup, mean AgNOR number of group and mean TAA/TNA ratio of these groups were given in Table 1. 

**Figure 2:**
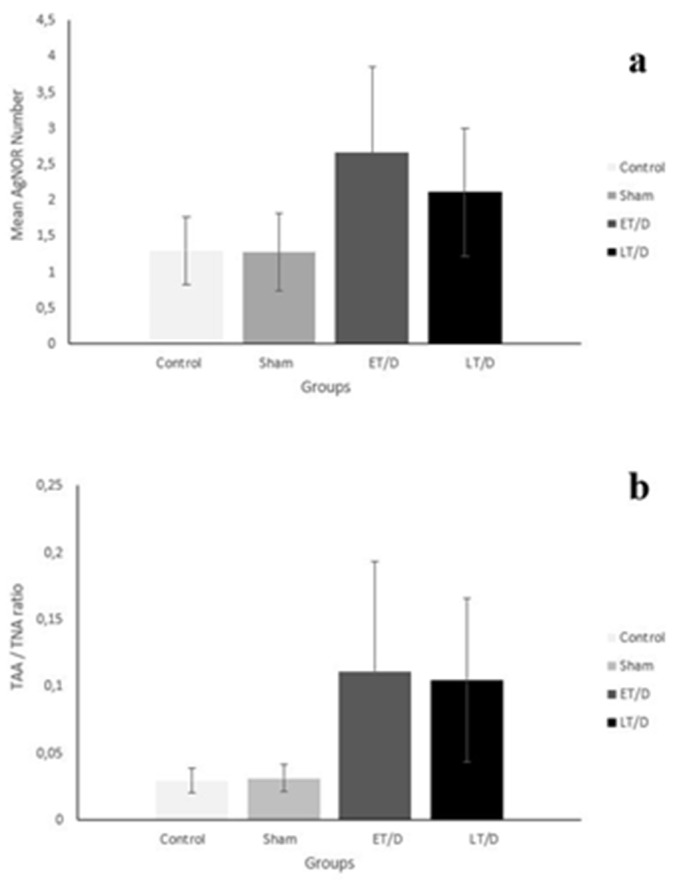
Comparison of mean AgNOR number (**a**) and TAA/TNA ratio (**b**) of groups.

When all groups to be taken into consideration, the differences were statistically significant among the groups for both mean AgNOR number (χ^2^ = 355.977, *p* < 0.001) and TAA/TNA (χ^2^ = 730.555, *p* < 0.001), respectively (Table 1 and Figure 2). In order to understand the causes of these differences from which groups, double comparison of the groups was carried out.

When the two groups were compared in terms of mean AgNOR number, there were statistically significant differences between control and early T/D (*Z* = −14.086, *p* < 0.001), between control and late T/D (*Z* = −11.534, *p* < 0.001), between sham and early T/D (*Z* = −14.348, *p* < 0.001), between sham and late T/D (*Z* = −12.026, *p* < 0.001) and between early T/D and late T/D (*Z* = −5.480, *p* < 0.001). Conversely no significant difference between control and sham was detected (*Z* = −1.299, *p* = 0.194) (Table 2).

When the two groups were compared in terms of TAA/TNA, there were statistically significant differences between control and early T/D (*Z* = −19.227, *p* < 0.001), between control and late T + D (*Z* = −18.949, *p* < 0.001), between sham and early T/D (*Z* = −19.267, *p* < 0.001) and between sham and late T/D (*Z* = −18.948, *p* < 0.001). But there were no significant differences between control and sham (*Z* = −1.280, *p* = 0.201) and between early T/D and late T/D (*Z* = −0.356, *p* = 0.722) for TAA/TNA (Table 3).

When the ANPCT and PAT values to be considered, there were statistically significant differences among the groups for ANPCT (χ^2^ = 11.031, *p* = 0.012) and PAT (χ^2^ = 9.798, *p* = 0.020), respectively (Table 4). To understand the causes of these differences among groups, double comparison was carried out. When the two groups were compared in terms of ANPCT, there were statistically significant differences between control and early T/D (*Z* = −2.611, *p* = 0.009), between sham and early T/D (*Z* = −2.619, *p* = 0.009). Conversely there were no significant difference between control and sham (Z = −1.471, *p* = 0.141), between sham, late T/D (*Z* = −1.571, *p* = 0.116), and between early T/D and late T/D (*Z* = −0.94, *p* = 0.347). When the two groups were compared in terms of PAT, there were statistically significant differences between control and early T/D (*Z* = −2.611, *p* = 0.009) and between sham and early T/D (*Z* = −2.402, *p* = 0.016). But there were no significant differences between control and sham (*Z* = −0.319, *p* = 0.750), between control and late T/D (*Z* = −1.781, *p* = 0.075), between sham and late T/D (*Z* = −1.776, *p* = 0.076) and between early T/D and late T/D (*Z* = −0.104, *p* = 0.917).

**Figure 3:**
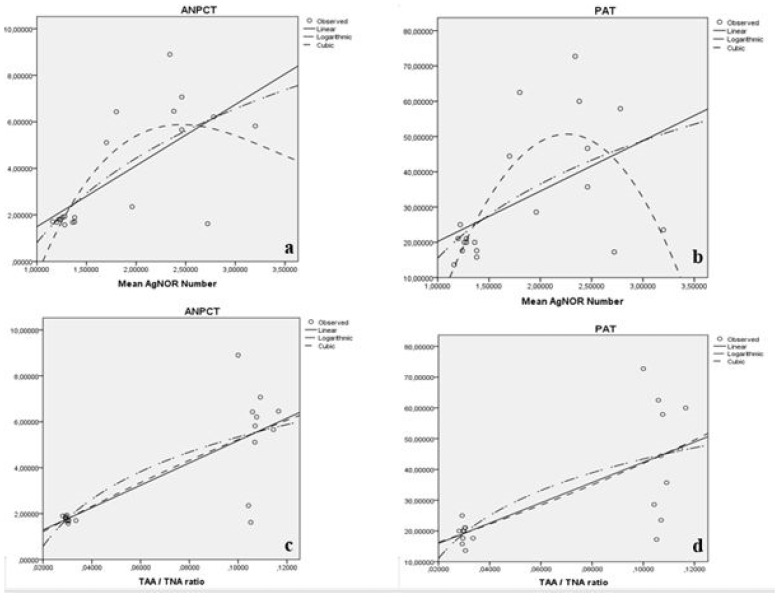
Relation between mean AgNOR numbers of testes cells and ANPCT (**a**), between mean AgNOR numbers of testes cells and PAT (**b**). Relation between mean TAA/TNA ratio of testes cells and ANPCT (**c**) and between TAA/TNA ratio of testes cells and PAT (**d**).

When we performed the polynomial regression analysis, statistically significant relation between mean AgNOR numbers of testes cells and both ANPCT and PAT were detected (*p* < 0.05) (Table 5, Figure 3). Additionally, statistically significant relation between TAA/TNA ratio of testes cells and both ANPCT and PAT were detected (*p* < 0.05) (Table 5, Figure 3).

**Figure 4:**
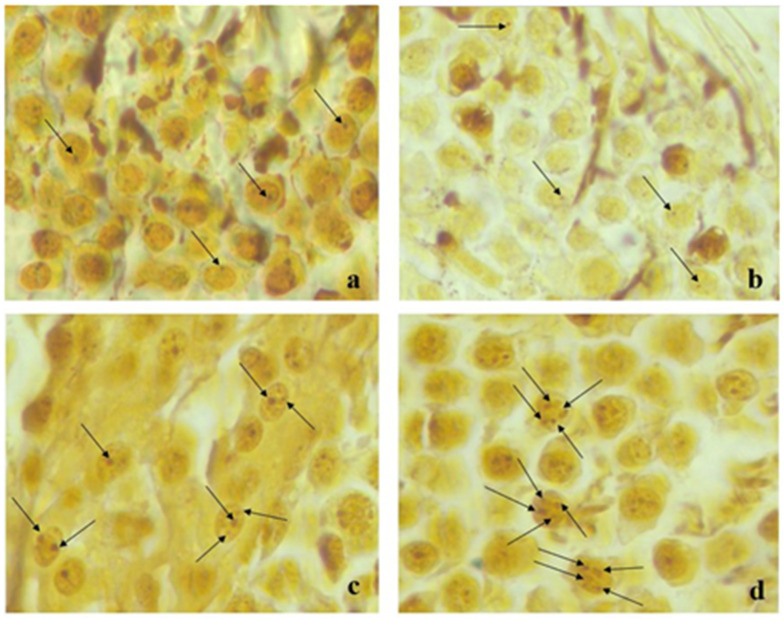
Representative images of silver stained NOR (**a**) Control, (**b**) Sham, (**c**) Early T/D, (**d**) Late T/D for testicular cells. The arrows showed the AgNOR proteins.

Representative images of silver stained NOR a: Control, b: Sham, c: Late T/D, d: Early T/D for testicular cells were given in the Figure 4.

## 4. Discussion

Ischemic injury to the testicle is a complex event, encountered with hypoxia and acidosis, frequently seen in pediatric surgery and can cause structural and functional damage of the reproductive tissues and cells. Therefore, obtaining early information about the duration and severity of ischemic damage is of great importance for the development of an effective treatment strategy. We present the findings of our study designed for this purpose in the light of the literature.

Alterations of AgNOR proteins’ shape, number, and distribution reflect the metabolic activities and protein synthesis capacity of the cells. AgNOR proteins give information about the proliferation index of different cells. Especially more certain information about the proliferative and metabolic activity of the different cells and response to dangerous agents may be obtained via detection of the TAA/TNA ratio. Moreover, this technique is inexpensive and easy to perform. Detection and description of new biological markers to get information about the cellular response to dangerous agents such as hypoxic conditions caused by ischemia is important to improve the diagnostic accuracy. We performed different studies to detect the effects and role of AgNOR proteins in human hair loss [8], hair root cells of humans at different age and sex [9], buccal epithelial cells of healthy persons [10], carbon monoxide (CO) intoxication of lungs [11], the effect of acute CO intoxication on cardiac damage [12], chronic CO exposure in rat myocardium [13], femoral muscle cells of rats exposed CO gas [14], use of AgNOR in skeletal muscle cells for prediction of chronic CO exposure [15], fine needle aspiration (FNA) samples of thyroid [16], benign thyroid nodules and normal thyroid tissue [17], determinations in nondiagnostic FNA samples of thyroid nodules [18], cytologic discrimination of follicular thyroid lesions [19], clinical exacerbation of chronic obstructive pulmonary disease [20], renal Ischemia/reperfusion (I/R) injury [21], the effect of capsaicin in human colon adenocarcinoma [26], for detecting the most reliable dose of rhamnetin [27] in cancer treatments [28]. To our knowledge, the present study was carried out for the first time on the AgNOR in ischemic damage of rat testis.

In our previous study, we detected that the level of the AgNOR proteins increased depending on the CO exposure (a cause of the hypoxic condition). We detected that TAA/NA ratio can be used as a marker for obtaining knowledge about the level of myocardial damage instead of histopathological evaluation scores in rats exposed to CO gas chronically (control, 1000 and 3000 ppm CO concentration with a flow rate of 4 L/min for 30 min/day for 7 days, respectively) [12]. Moreover, we detected that both the TAA/NA ratio and the mean AgNOR number provided information about the existence or absence of CO exposure chronically (1000 and 3000 ppm, respectively for 30 min a day for 7 d) and the TAA/NA ratio can be used as an indicator for detection of the CO exposure level that causes hypoxia [15]. In our previous studies, we reported that AgNOR protein may give information about the cardiomyopathy (CMY) levels and be used to detect the CO intoxication levels instead of carboxyhemoglobin (COHb) in later periods in the acute hypoxia condition caused by different densities of CO intoxication (1000, 3000, and 5000 ppm at a flow rate of 4 L/min for 30 min) [11].

In the current study, the differences were statistically significant among the groups for both mean AgNOR number and TAA/TNA. The differences between control and early T/D, between control and late T/D, between control and late T/D, between sham and early T/D, between sham and late T/D, and between early T/D and late T/D were statistically significant for AgNOR amount. All alive cells must protect themselves from internal and external dangerous agents such as ischemic injury. May these proteins occur against ischemic injury as a protective or trigger the occurring other proteins that have a protective effect on the regulation of gene expression and signaling transduction pathways in the ischemic injury? May these proteins be used as therapeutic agents to prevent the negative effects of ischemic injury? Perhaps these conditions may lead to the development of new therapeutic approaches in the near future. In order to reveal this situation more clearly, additional studies should be performed on the current topic.

Although emergency treatment is essential for the rapid recovery of testicular tissue from ischemia, it is equally important to develop postoperative strategies to avoid the negative effects of reperfusion. Although clinicians have been producing projects on this subject for a long time, they have not been able to implement an effective method, probably due to the limited clinical evaluation. Another point that the authors have in common is that the apoptosis mechanism that occurs as well as the reperfusion damage also causes more harm than benefit. When cells are seen to end their life by themselves through an apoptotic mechanism, they show some biochemical (fragmentation of DNA, phosphatidylserine translocated to the outer leaflet membrane of apoptotic cells) and morphological changes (cell shrinkage, chromatin condensation, apoptotic bodies) that lead to apoptosis. Various methods can be used to evaluate apoptosis after ischemia. DNA fragmentation can be demonstrated by TUNEL or agarose gel electrophoresis and ELISA methods. Similarly, transmission electron microscopy is an appropriate method for determining morphological changes. Moreover, apoptosis can be identified using the caspase-3 method. However, these methods can be complex, be economically high, and they also have negative features in terms of time and speed.

In addition, the apoptotic index gives the ratio of cells that are damaged and will die in a programmed way and remaining intact cells, rather than showing the viability of the returning testicle. However, by measuring regenerated/proliferating cells with AgNOR, we can show not only survivors but also cells that can repair themselves. This may be a more valid method than the apoptotic index in demonstrating treatment efficacy in experimental studies. There is no study showing the relationship between AgNOR and apoptotic index. This study examines whether AgNOR proteins can be used instead of the TUNEL procedure that shows apoptotic index in the evaluation of testicular ischemia, and it is indicated that they can be used in the evaluation of the potential of therapeutic approaches that can be used against testicular damage.

It has been presented that only the AgNOR staining procedure can be used to detect lung [11] and myocardial [12,13] damage instead of histopathological evaluation scores. In the current study, statistically significant differences among the groups for ANPCT and PAT were detected. The differences between control and early T/D, between control and late T/D, between sham and early T/D were statistically significant for ANPCT. There were also statistically significant differences between control and early T/D and between sham and early T/D for PAT. According to the polynomial regression analysis, a statistically significant relation between both mean AgNOR numbers and TAA/TNA ratio of testes cells and both ANPCT and PAT were detected. So, it may be said that the AgNOR proteins amount may give information about the levels of the testes injury after T/D process.

One of the biggest problems for surgeons in torsion surgeries is the possibility of evaluating whether the testicular tissue is completely necrotic or can be revitalized. Completely dying tissue not only does not regenerate but also damages the opposite testicle with the formation of antibodies. Thus, leaving the necrotic testicle in place may contribute to infertility through antibody formation. On the other hand, removing the testicles that have the capacity to revive is also a bigger problem. Considering that Color Doppler ultrasound sensitivity is about 70% [29], the medical and legal consequences of removing intact testicular tissue can be troublesome for surgeons. Biopsy of testicular tissue during torsion surgery may be ethically objectionable, but if there is, appendix testicular tissue can be removed or a thin needle biopsy from the testicle itself can be taken more ethically and harmlessly. Thus, the measurement made with AgNOR can give us objective information about whether the testicle will return or not. Since testicular biopsy is not included in routine clinical practice in testicular torsion cases and it is not directly related to the subject of the article, we added it as a recommendation. However, as the research on the AgNOR application increases, we think that biopsy will be added to the treatment as a clinical application in these cases.

AgNOR can be used as a marker of the proliferation index and metabolic activity of cells. By looking at these values, information can be obtained about the revival potential or survival potential of the cells. The increase in AgNOR levels is to maintain homeostasis, and the values of these proteins vary depending on the type and amount of potentially dangerous agents or conditions. This process continues until the balance of the cells is restored and returns to the control group values when homeostasis is achieved. In pathological conditions, the survival rate decreases if AgNOR levels fall below the control group value where physiological conditions prevail. 

One of the biggest advantages of this method is that it is more reliable than the TUNEL method, which has individual interpretation differences since the evaluation results are obtained with the help of a program [25]. It gives evaluation results with high specificity and sensitivity.

## 5. Conclusions

It may be said that AgNOR proteins are usable for the early observation of ischemic injury levels. The amount of AgNOR protein can also enlighten us about the extent of testicular damage after T/D treatment. Evaluation of changes in shape, number, distribution, and quantity of these proteins can provide information on the duration, density, and damage of T/D. It may also help the physician in the development of effective treatment strategies for cases. The AgNOR staining technique is a simple, cheap, reliable marker for the evaluation of ischemic injury. Perhaps with this approach, new therapeutic methods can be developed in the near future.

## Figures and Tables

**Table 1:** Mean AgNOR number and TAA/TNA ratio of each subgroup, average AgNOR number of group and average TAA/TNA ratio of these groups.

Groups	Mean AgNOR Number ± SD (*n* = 50)	Mean TAA/TNA ± SD (*n* = 50)	Average AgNOR Number of Group/Median (Range) (*n* = 250)	Mean TAA/TNA of Group/Median (Range) (*n* = 250)	χ^2^	*p*
C1	1.28 ± 0.536	0.03 ± 0.008	1.288 ± 0.471/1 (2)	0.029 ± 0.009/0.028 (0.093)	355.977 * 730.555 ^&^	0.000 * 0.000 ^&^
C2	1.38 ± 0.490	0.029 ± 0.007				
C3	1.28 ± 0.454	0.03 ± 0.012				
C4	1.24 ± 0.431	0.03 ± 0.009				
C5	1.26 ± 0.443	0.028 ± 0.008				
S1	1.22 ± 0.419	0.029 ± 0.009	1.264 ± 0.540/1(3)	0.031 ± 0.01/0.03 (0.056)		
S2	1.36 ± 0.663	0.03 ± 0.012				
S3	1.2 ± 0.404	0.03 ± 0.011				
S4	1.38 ± 0.697	0.034 ± 0.011				
S5	1.16 ± 0.422	0.031 ± 0.009				
ET/D 1	2.46 ± 1.147	0.109 ± 0.028	2.656 ± 1.183/3 (6)	0.111 ± 0.082/0.098 (0.919)		
ET/D 2	3.2 ± 1.212	0.107 ± 0.032				
ET/D 3	2.78 ± 1.298	0.108 ± 0.033				
ET/D 4	2.46 ± 1.073	0.115 ± 0.125				
ET/D 5	2.38 ± 1.008	0.117 ± 0.125				
LT/D 1	1.96 ± 0.88	0.104 ± 0.023	2.104 ± 0.89/2 (4)	0.104 ± 0.061/0.103 (0.96)		
LT/D 2	2.72 ± 1.011	0.105 ± 0.029				
LT/D 3	2.34 ± 0.798	0.1 ± 0.028				
LT/D 4	1.8 ± 0.606	0.106 ± 0.025				
LT/D 5	1.7 ± 0.707	0.107 ± 0.127				

C: control; S: sham; ET/D: early torsion/detorsion group; LT/D: late torsion/detorsion group; *: For mean AgNOR number; ^&^ = For TAA/TNA; TAA/TNA: total AgNOR area/total nuclear area.

**Table 2:** Binary comparison results of the groups for mean AgNOR number.

Groups	C		S		ET/D		LT/D	
	*Z*	*p*	*Z*	*p*	*Z*	*p*	*Z*	*p*
C			−1.299	0.194	−14.086	0.000	−11.534	0.000
S	−1.299	0.194			−14.348	0.000	−12.026	0.000
ET/D	−14.086	0.000	−14.348	0.000			−5.480	0.000
LT/D	−11.534	0.000	−12.026	0.000	−5.480	0.000		

C: control; S: sham; ET/D: early torsion/detorsion group; LT/D: late torsion/detorsion group.

**Table 3:** Binary comparison results of the groups for TAA/TNA ratio.

Groups	C		S		ET/D		LT/D	
	*Z*	*p*	*Z*	*p*	*Z*	*p*	*Z*	*p*
C			−1.280	0.201	−19.227	0.001	−18.948	0.001
S	−1.280	0.201			−19.267	0.001	−18.948	0.001
ET/D	−19.227	0.001	−19.267	0.001			−0.356	0.722
LT/D	−18.948	0.001	−18.948	0.001	−0.356	0.722		

C: control; S: sham; ET/D: early torsion/detorsion group; LT/D: late torsion/detorsion group.

**Table 4:** The comparison of ANPCT and PAT in all groups.

Groups	ANPCT	Mean ± SD/Median (Range) for ANPCT	PAT	Mean ± SD/Median (Range) for PAT	χ^2^	*p*
C1	1.93	1.82 ± 0.147/1.89 (0.36)	20.00	18.894 ± 2.143/20 (5.27)	11.031 ^*^ 9.798 ^&^	0.012 ^*^ 0.020 ^&^
C2	1.89		15.78			
C3	1.57		21.05			
C4	1.81		17.64			
C5	1.90		20.00			
S1	1.81	1.716 ± 0.054/1.7 (0.13)	25.00	19.464 ± 4.208/20 (11.37)		
S2	1.68		20.00			
S3	1.68		21.05			
S4	1.70		17.64			
S5	1.71		13.63			
ET/D 1	7.07	6.244 ± 0.559/6.21 (1.41)	35.71	44.756 ± 15.34/46.66 (36.48)		
ET/D 1	5.82		23.52			
ET/D 1	6.21		57.89			
ET/D 1	5.66		46.66			
ET/D 1	6.46		60.00			
LT/D 1	2.35	4.882 ± 2.985/5.11 (7.28)	28.57	45.096 ± 23/44.44 (55.49)		
LT/D 2	1.62		17.24			
LT/D 3	8.90		72.73			
LT/D 4	6.43		62.50			
LT/D 5	5.11		44.44			

C: control; S: sham; ET/D: early torsion/detorsion group; LT/D: late torsion/detorsion group; ANPCT: average number of apoptotic cells per tubule; PAT: percentage of apoptotic tubule values; *: For ANPCT; ^&^: For PAT.

**Table 5:** Model summary and parameter estimates for AgNOR numbers, TAA/TNA, and antioxidant enzymes.

		Model Summery					Parameter Estimates			
Variable	Equation	R^2^	F	df1	df2	sig	Constant	b1	b2	b3
M-AgNOR-N and ANPCT	Linear	0.491	17.335	1	18	0.001	−1.135	2.626		
	Log	0.540	21.120	1	18	0.000	0.805	5.240		
	Cubic	0.612	8.419	3	16	0.001	−16.973	23.016	−7.378	0.731
M-AgNOR-N and PAT	Linear	0.255	6.162	1	18	0.023	5.953	14.277		
	Log	0.318	8.385	1	18	0.010	15.506	30.314		
	Cubic	0.568	7.009	3	16	0.003	−104.265	133.132	−26.750	−0.807
TAA/TNA and ANPCT	Linear	0.632	30.926	1	18	0.000	0.317	48.638		
	Log	0.631	30.803	1	18	0.000	12.153	2.959		
	Cubic	0.632	14.609	2	17	0.000	0.052	59.827	−80.823	0.000
TAA/TNA and PAT	Linear	0.511	18.810	1	18	0.000	9.351	329.732		
	Log	0.510	18.705	1	18	0.000	89.558	20.050		
	Cubic	0.511	8.885	2	17	0.002	10.496	289.733	0.000	2509.205

M-AgNOR-N: mean AgNOR number; Log: logarithmic; TAA/TNA: total AgNOR area/total nuclear area; ANPCT: average number of apoptotic cells per tubule; PAT: percentage of apoptotic tubule values.

## Data Availability

The data presented in this study are available on reasonable request from the corresponding author.

## Abbreviations

T/D	torsion-detorsion
I-R	ischemia-reperfusion
DNA	Deoxyribonucleic acid
ELISA	Enzyme-Linked ImmunoSorbent Assay
TUNEL	Terminal deoxynucleotidyl transferase nick end labeling method
NORs	Nucleolar-organizing regions
AgNOR	Post-silver staining NORs
TAA	total AgNOR area
TNA	total nuclear area
i.p.	intraperitoneal injection
ANPCT	average number of apoptotic cells per tubule
PAT	percentage of apoptotic tubule values
SD	standard deviation
ET/D	early torsion-detorsion group
LT/D	late torsion-detorsion group
